# The therapeutic potential of antioxidants, ER chaperones, NO and H_2_S donors, and statins for treatment of preeclampsia

**DOI:** 10.3389/fphar.2014.00119

**Published:** 2014-05-27

**Authors:** Tereza Cindrova-Davies

**Affiliations:** Centre for Trophoblast Research and Department of Physiology, Development and Neuroscience, University of CambridgeCambridge, UK

**Keywords:** preeclampsia, angiogenesis, antioxidants, ER chaperones, NO, H_2_S, statins

## Abstract

Preeclampsia is a complex multifactorial disease. Placental oxidative stress, a result of deficient spiral artery remodeling, plays an important role in the pathophysiology of preeclampsia. Antiangiogenic factors secreted from malperfused placenta are instrumental in mediating maternal endothelial dysfunction and consequent symptoms of preeclampsia; the mechanism is likely to involve increased ET-1 secretion and reduced NO bioavailability. Therapeutic interventions so far remain only experimental and there is no established remedy for the treatment of preeclampsia. This review concentrates on the evidence for the therapeutic potential of antioxidants, ER chaperones, NO and H_2_S donors, and statins. These compounds display pleitropic antioxidant, anti-inflammatory, and pro-angiogenic effects in animal and *in vitro* studies. Although clinical trials on the use of antioxidant vitamins in pregnancy proved largely unsuccessful, the scope for their use still exists given the beneficial cardioprotective effects of antioxidant-rich Mediterranean diet, periconceptual vitamin use and the synergistic effect of vitamin C and L-arginine. Encouraging clinical evidence exists for the use of NO donors, and a clinical trial is underway testing the effect of statins in treatment of preeclampsia. H_2_S recently emerged as a novel therapeutic agent for cardiovascular disease, and its beneficial effects were also tested in animal models of preeclampsia. It is risky to prescribe any medication to pregnant women on a large scale, and any future therapeutic intervention has to be well tested and safe. Many of the compounds discussed could be potential candidates.

## THE SYNDROME OF PREECLAMPSIA

Preeclampsia affects 3–5% of all pregnancies. The traditional definition of preeclampsia as *de novo* onset of hypertension and proteinuria after 20 weeks of gestation has been recently modified by the American College of Obstetricians and Gynecologists in recognition of the syndromic nature of preeclampsia. In the absence of proteinuria, preeclampsia is diagnosed as hypertension in association with thrombocytopenia, impaired liver function, new development of renal insufficiency, pulmonary edema, or new-onset cerebral or visual disturbances (American College of Obstetricians Gynecologists [ACOG], 2013). Hypertension, proteinuria, edema, and other systemic manifestations of the syndrome of preeclampsia are the direct consequences of maternal endothelial dysfunction. The syndrome is a major cause of maternal and fetal morbidity and mortality, and results in mild to severe microangiopathy of target organs, including the brain, liver, kidney, and placenta (Sibai et al., 2005). Severe maternal complications include seizures (eclampsia), stroke, renal failure, liver failure, and/or rupture, HELLP syndrome (hemolysis, elevated liver enzymes, and low platelets), and death (Young et al., 2010). Fetal complications include prematurity due to preterm delivery, fetal growth restriction, fetal/neonatal hypoxic neurological injury, perinatal death, and long-term cardiovascular morbidity associated with low birth weight (Sibai et al., 2005).

Preeclampsia (PE) is a complex multifactorial disease; many factors, including genetic predisposition, immunological interactions, maternal endothelial function and environmental factors interact and culminate in the disease manifestation. In recent years, PE is commonly subdivided into early and late-onset, as the two conditions seem to have different underlying etiologies. Both early and late onset diseases present with abnormal placental perfusion and oxidative stress, however, poor placentation and deficient conversion of maternal spiral arteries only underlie the pathology of the early onset disease, whilst the late onset condition is associated with normal uterine spiral artery conversion and normally grown baby (Huppertz, 2008). Redman recently proposed a novel hypothesis for the pathology of late-onset PE, suggesting an intrinsic cause due to microvillous overcrowding, as placental growth reaches its functional limit. In both conditions, oxidatively stressed syncytiotrophoblast over-secrets proteins that perturb maternal angiogenic balance (Redman et al., 2014). Abnormal placentation is recognized as a main prerequisite for the pathogenesis of early onset preeclampsia. The disease seems to progress in two stages; in stage I (preclinical), poor development of the early placenta leads to deficient remodeling of maternal spiral arteries and consequent ischemia-reperfusion type injury of the placenta and oxidative stress. In stage II (clinical), the ischemic/malperfused placenta triggers the secretion of a mixture of placental factors, including antiangiogenic factors, proinflammatory cytokines and apoptotic debris that culminate in an enhanced maternal inflammatory response and in turn induce systemic endothelial dysfunction and the maternal syndrome of preeclampsia (Roberts and Cooper, 2001; Redman and Sargent, 2005).

In conditions of PE and IUGR which result in fetal hypoxia, there is a tendency to refer to fetoplacental hypoxia as the underlying cause. This concept has been challenged by several groups who advocate caution when interpreting results from pregnancies complicated by PE and IUGR (Kingdom and Kaufmann, 1997; Mayhew et al., 2004; Huppertz et al., 2014). These studies suggest that late-onset PE and late onset IUGR with present end diastolic flow (EDF) are associated with ischemic uteroplacental hypoxia in which the delivery of blood to the intervillous space is compromised due to deficient trophoblast invasion. This placental malperfusion results in ischemia-reperfusion injury affecting trophoblast and vascular endothelium, however fetal extraction of O_2_ is not compromised and there is evidence of increased placental branching angiogenesis (Mayhew et al., 2004). In contrast, pregnancies complicated by early onset PE, which is usually accompanied with early onset IUGR with absent/reversed EDF are often referred to as ‘placental hyperoxia’ due to compromised fetoplacental blood flow which fails to extract oxygen from the intervillous space, leading to poor fetal oxygenation and higher than normal intervillous oxygen levels (postplacental hypoxia; Kingdom and Kaufmann, 1997; Mayhew et al., 2004).

Medical intervention in preeclampsia remains limited, centering on management of maternal hypertension and systemic complications, seizure prevention with magnesium sulfate, and delivering the fetus before term as a measure of treating maternal effects and preventing further growth restriction. However, any benefits this confers are offset by the complications of prematurity. Hence, identifying interventions that would modulate the pathological processes involved in the disease pathogenesis are key to intervening effectively in preeclampsia to improve maternal and fetal prognosis. This review will focus on the role of angiogenic and antiangiogenic factors in mediating maternal endothelial dysfunction and explore some therapeutic strategies aimed at targeting these factors in order to reverse/ameliorate the disease pathology.

## EVIDENCE FOR THE ROLE OF ANGIOGENIC FACTORS IN PREECLAMPSIA

Placental vasculature is plastic and changes constantly throughout pregnancy to facilitate the rapid growth, and supply the increasing metabolic needs of the growing fetus (Charnock-Jones et al., 2004). Normal placental development is regulated by proangiogenic and antiangiogenic factors. The processes of vasculogenesis, *de novo* formation of blood vessels from precursor cells, and angiogenesis, creation of vessels from pre-existing vessels, are critical in the fetal and placental development for effective transport of oxygen, nutrients and fetal growth and development (Charnock-Jones et al., 2004). In contrast, angiogenesis is a rare event in adult vascular networks under physiological conditions, being limited to organs of the female reproductive tract (endometrium, ovary, placenta). Appearance of new blood vessel growth in other organs is regarded as pathological, and can be involved in conditions such as wound healing, tumor growth, and retinopathy.

Placental abnormalities lead to secretion of a mixture of proteins that stimulate maternal endothelium and result in the disease presentation. Recently, circulating antiangiogenic proteins, soluble vascular endothelial growth factor receptor 1 [VEGF-R1, also referred to as soluble fms-like tyrosine kinase (sFlt1)] and soluble TGF-β co-receptor endoglin (sEng), have been implicated in the pathogenesis of many of the maternal features of preeclampsia. We will first consider the role of proangiogenic factors, VEGF and transforming growth factor (TGF-β), and their receptors in vascular homeostasis (**Table 1**). VEGFs are a family of secreted dimeric glycoproteins involved in vasculogenesis and angiogenesis. In humans and other mammals, VEGF-A (referred here as VEGF) and its placental homologue placental growth factor (PlGF) are the most important members with proangiogenic activity (Keck et al., 1989; Leung et al., 1989; Maglione et al., 1991). VEGF promotes survival and proliferation of endothelial cells and induces vascular permeability (Keck et al., 1989; Leung et al., 1989). Two VEGF receptors, VEGF-R1 (Flt-1) and VEGF-R2 (Flk-1/KDR) are present on endothelial cells. VEGF binds to both receptors whilst PlGF homodimers bind exclusively to VEGF-R1. VEGF-R2 it thought to primarily mediate the actions of VEGF on endothelial cells. The function of VEGF-R1 is less well defined, although it is thought to modulate VEGF-R2 signaling. Another function of VEGF-R1 is to act as a decoy receptor (sFlt-1), sequestering VEGF from VEGF-R2 binding (**Table 1**; Park et al., 1994; Waltenberger et al., 1994; Keyt et al., 1996). TGF-β is another important molecule involved in angiogenesis, it has been implicated in the development of the vasculature (Oshima et al., 1996). TGF-β signals via binding to both type I (ALK1 and ALK5) and type II receptors. In addition, TGF-β signaling in the vasculature involves coreceptors that act to modulate its effects. Endoglin (CD105) is a TGF-β coreceptor expressed by endothelial cells and placental syncytiotrophoblast. TGF-β effects *in vitro* seem concentration-dependent, inducing proliferation and migration of endothelial cells at low doses (ALK1 mediated), and inhibiting proliferation and migration of endothelial cells at high doses (via ALK5; Goumans et al., 2002). In addition, TGF-β is an important modulator of VEGF signaling, further linking TGF-β to angiogenesis (**Table 1**; Shao et al., 2009).

**Table 1:** A brief description of function of angiogenic and antiangiogenic growth factors and their receptors in pregnancy.

Molecules	Function and changes in pathological conditions
Angiogenic	Factors
VEGF-A	Potent angiogenic protein and trophic cytokine essential for endothelial integrity
	Free VEGF concentration decreased in preeclamptic women at disease presentation and even before the onset of disease (Maynard et al., 2003; Taylor et al., 2003; Levine et al., 2004)
PlGF	Key molecule in angiogenesis and vasculogenesis, in particular during embryogenesis. The main source of PlGF during pregnancy is the placental trophoblast
	Reduced in maternal plasma in preeclampsia (Maynard et al., 2003; Taylor et al., 2003; Levine et al., 2004)
TGF-β	Important molecule involved in angiogenesis, it has been implicated in the development of the vasculature (Oshima et al., 1996). *In vitro* effects are concentration-dependent, inducing proliferation and migration of endothelial cells at low doses (ALK1 mediated), and inhibiting proliferation and migration of endothelial cells at high doses (via ALK5; Goumans et al., 2002). Important modulator of VEGF signaling, further linking TGF-β to angiogenesis (Shao et al., 2009)

**Receptors**	
VEGF-R1 (Flt-1)	All members of the VEGF family stimulate cellular responses by binding to tyrosine kinase receptors (the VEGF-Rs) on the cell surface, causing them to dimerize and become activated through transphosphorylation
VEGF-R2 (Flk-1)	VEGF-A binds to VEGF-R1 (Flt-1) and VEGF-R2 (KDR/Flk-1), whilst PlGF only binds to VEGF-R1. VEGF-R2 appears to mediate almost all of the known cellular responses to VEGF. The function of VEGF-R1 is less well defined, although it is thought to modulate VEGF-R2 signaling. Another function of VEGFR-1 is to act as a decoy receptor (sFlt-1), sequestering VEGF from VEGF-R2 binding (Park et al., 1994; Waltenberger et al., 1994; Keyt et al., 1996)
TGF-β RI (ALK1,5) TGF-β RII Endoglin (CD105)	TGF-β signals via binding to both type I (ALK1 and ALK5) and type II receptors. In addition, TGF-β signaling in the vasculature involves coreceptors, e.g., endoglin (CD105), that act to modulate its effects. Endoglin is a membrane glycoprotein expressed by endothelial cells and placental syncytiotrophoblast. It has a crucial role in angiogenesis, therefore, making it an important protein for tumor growth, survival and metastasis of cancer cells to other locations in the body (Goumans et al., 2002; Shao et al., 2009).

**Antiangiogenic**	**Factors**

sFlt-1	Formed by alternative splicing of the pre-mRNA encoding VEGF-R1. It can bind circulating VEGF and PlGF as it lacks the cytoplasmic and transmembrane domain, thus preventing their interactions with endogenous receptors. Increased in maternal plasma in preeclampsia (Taylor et al., 2003; Levine et al., 2004,^2006^; Chaiworapongsa et al., 2005; Steinberg et al., 2009)
sEng	Reduces endothelial tube formation in vitro and leads to increased capillary permeability. It blocks TGF-β 1-induced vasodilation through interference of TGF-β 1 binding to its receptor (Venkatesha et al., 2006). sEng is elevated in the sera of preeclamptic women, its circulating levels increase markedly beginning 2–3 months before the onset of preeclampsia, concentrations correlating with disease severity (Levine et al., 2006)

The vascular endothelium relies on proangiogenic factors. Release of placentally derived antiangiogenic factors into maternal circulation is thus likely to cause angiogenic factor imbalance, leading to endothelial dysfunction that manifests as the symptoms of preeclampsia. Two such factors, sFlt-1 and sEng, have been implicated in the pathophysiology of preeclampsia (**Table 1**). Soluble Flt-1 is formed by alternative splicing of the pre-mRNA encoding VEGF-R1. It can bind circulating VEGF and PlGF as it lacks the cytoplasmic and transmembrane domain, thus preventing their interactions with endogenous receptors. Soluble endoglin receptor sEng reduces endothelial tube formation *in vitro* and leads to increased capillary permeability. It blocks TGF-β1-induced vasodilation through interference of TGF-β1 binding to its receptor, this effect is likely to involve NO (Venkatesha et al., 2006).

Let us now consider the clinical and experimental evidence for the role of angiogenic factor imbalance in the pathophysiology of preeclampsia. The circulating levels of two placental-derived antiangiogenic factors, sFlt-1 and sEng, are elevated in the circulation of women with preeclampsia, and may begin to rise even before the onset of clinical symptoms, whereas the circulating concentrations of VEGF and PlGF are reduced in preeclamptic women at disease presentation and even before the onset of clinical symptoms (Taylor et al., 2003; Levine et al., 2004, 2006; Chaiworapongsa et al., 2005; Steinberg et al., 2009). The levels of circulating sFlt-1 increase and PlGF decrease during the last two months of pregnancy in normotensive women, however, these changes are significantly more pronounced in women who later develop preeclampsia and occur on average about 5 weeks before the onset of the disease (Levine et al., 2004). sEng is elevated in the sera of preeclamptic women, its circulating levels increase markedly beginning 2–3 months before the onset of preeclampsia, concentrations correlating with disease severity (Levine et al., 2006). However, it should be noted that despite the strong evidence linking angiogenic factor imbalance with PE, not all preeclamptic patients present with increased sFlt-1 and decreased VEGF/PlGF levels, and other mechanisms such as inflammation, oxidative stress, immunological interactions, maternal endothelial function, etc. also play an important role in the pathophysiology of this multifactorial disease.

Animal studies provide strong evidence linking sFlt-1 to the pathogenesis of preeclampsia. Although animal models do not develop preeclampsia, exogenous administration of sFlt-1 to pregnant rats induces some of the symptoms similar to the preeclamptic phenotype, including hypertension, proteinuria, and glomerular endotheliosis (Maynard et al., 2003). Similarly, glomerular endotheliosis and proteinuria can be induced in non-pregnant mice using antibodies against VEGF (Sugimoto et al., 2003), or by reducing glomerular VEGF levels by 50% (Eremina et al., 2003). In addition, administration of sFlt-1 can block VEGF and PlGF-induced microvascular relaxation of rat renal arterioles *in vitro* (Maynard et al., 2003). These studies all demonstrate that abnormal inhibition of endogenous VEGF activity can induce maternal preeclampsia-like symptoms. Similarly, administration of a therapeutic VEGF neutralizing antibody leads to renal pathology which can be replicated in adult animals by conditional gene targeting (Eremina et al., 2008). sFlt-treated animals do not develop additional symptoms of PE such as hemolysis and thrombocytopenia, seen in the HELLP syndrome. However, co-administration of sFlt-1 with sEng amplifies the symptoms, resulting in severe PE and/or HELLP syndrome, suggesting that these two placenta-derived factors could act in concert to induce severe preeclampsia (Venkatesha et al., 2006). We have shown similar synergistic effects between sFlt-1 and TNF-α. sFlt-1 sensitized endothelial cells to pro-inflammatory factors, increasing endothelial cell activation, as measured by increases in endothelial intercellular adhesion molecule 1 (ICAM1), vascular cell adhesion molecule 1, endothelin 1 (ET-1), von Willebrand factor and leukocyte adhesion, and led to reduction of AKT Ser^473^ and endothelial nitric oxide synthase (eNOS) Ser^1177^ phosphorylation (Cindrova-Davies et al., 2011). The survival of endothelial cells *in vivo* under non-pathological conditions is critically dependent on autocrine VEGF signaling. Endothelial specific ablation of VEGF results in progressive endothelial degeneration and sudden death of mutant animals (Lee et al., 2007), whilst autocrine VEGF is necessary for the survival of hematopoietic stem cells (Gerber et al., 2002), demonstrating that paracrine actions of VEGF are not sufficient to maintain the target cells. Thus, in preeclampsia the elevated circulating sFlt-1 is able to act in a dominant-negative fashion at the endothelial cell surface, blocking these autocrine signals (Cindrova-Davies et al., 2011).

## LINKING ANTIANGIOGENIC FACTORS WITH ENDOTHELIAL DYSFUNCTION

Maternal symptoms of preeclampsia present as a consequence of maternal endothelial cell dysfunction, not due to dysregulation of maternal angiogenesis. Secretion of antiangiogenic factors from the placenta can adversely affect maternal endothelial function. The higher relative concentrations of the antiangiogenic factors are thought to potentiate vascular endothelial cell injury in the liver, kidney, brain, and the placenta itself, and thus trigger the symptoms of preeclampsia. Placental oxidative stress due to malperfusion is now commonly considered as the underlying source for antiangiogenic factors which are at the root of the symptomatic phase of preeclampsia. However, the mechanism linking the angiogenic imbalance with endothelial dysfunction is still under investigation. Endothelin-1 (ET-1), a potent vasoconstrictor and pressor agent involved in the regulation of blood pressure, has been shown to play a crucial role in the development of hypertension in experimental animal models of placental hypoxia/ischemia (George and Granger, 2011). Long-term infusion of ET-1 into sheep also induced preeclampsia-like symptoms, i.e., hypertension, proteinuria, and decreased uteroplacental blood flow (Greenberg et al., 1997). In addition, several studies have reported elevated levels of plasma ET-1 in preeclamptic pregnant women, compared to controls (Taylor et al., 1990; Nova et al., 1991; Baksu et al., 2005).

The expression of VEGF and sFlt-1 can be regulated by the hypoxia-inducible factor-1 (HIF-1), a key component of a widely operative transcriptional response activated by hypoxia. Hypoxia increases HIF-1α stability, but it can also be up-regulated under non-hypoxic conditions by inflammatory cytokines or microtubule-depolymerising agents involving the NF-κB pathway (Jung et al., 2003). HIF-1-mediated sFlt-1 secretion can by induced in placental explants by hypoxia or hypoxia-reoxygenation (HR) *in vitro* (Achmad et al., 1997; Nagamatsu et al., 2004; Cindrova-Davies et al., 2007; Cudmore et al., 2007; Cindrova-Davies, 2009). These *in vitro* effects can be blocked by anti-oxidant vitamins, inhibitors of the p38 MAPK and NF-κB pathways, and by overexpression of the heme oxygenase (HO-1)/CO, suggesting the involvement of oxidative stress, NF-κB, p38 signaling and HO-1 in sFlt-1 and HIF-1α regulation (Cindrova-Davies, 2009). sEng inhibits TGF-β signaling in the vasculature, which includes effects on activation of eNOS and vasodilation. It has been postulated that increased circulating levels of sFlt-1 lead to reduction of VEGF and NOS, thus reducing NO production. Administration of sFlt-1 to pregnant rats induced hypertension and significantly increased the production of prepro-ET message levels in the renal cortex. The hypertensive response could be abolished with coadministration of an ET_A_ antagonist (Murphy et al., 2010). Administration of sFlt-1 to pregnant animals thus impairs VEGF-R2-mediated NO production, reducing NO bioavailability, thereby increasing systemic peripheral resistance and inducing hypertension. NO bioavailability may be further reduced by increased oxidative stress, and leads to enhanced ET-1 production and increased blood pressure (George and Granger, 2011).

Differences have been reported in the oxygen sensing ability of early vs. late onset PE. Rolfo et al. (2010) reported a disruption of oxygen sensing in early onset disease but not in the late-onset, and they speculate this contributes to decreased HIF-1α hydroxylation and breakdown, leading to its accumulation in early onset PE (Rolfo et al., 2010). As mentioned previously, hypoxia is disputed by many as the leading cause of placental pathologies. Instead, ischemia-reperfusion (Hung et al., 2002; Burton et al., 2009) or hyperoxia (Kingdom and Kaufmann, 1997; Huppertz et al., 2014) have been proposed as the underlying etiological factors in PE. Nevertheless, all these conditions lead to generation of reactive oxygen species (ROS), which can increase HIF-1α levels and stability. In addition, HIF-1α can be stabilized under normoxic conditions by other mechanisms such as components of the immune system, especially inflammatory cytokines, and hormones (Zhou and Brüne, 2006). Preeclampsia is a condition of excessive inflammatory responses mediated by syncytiotrophoblast microparticles, pathogens and DAMPs (damage-associated molecular pattern), which activate toll-like receptors (TLR) and through binding to immune cells promote persistent inflammatory conditions in this syndrome (Laresgoiti-Servitje, 2010). TNF-α can promote the release of sFlt-1 (Parrish et al., 2010), and similarly, AT1 autoantibodies can promote the secretion of sEng and sFlt-1 through TNF-α-mediated mechanisms (Zhou et al., 2008; Irani et al., 2010).

## OXIDATIVE STRESS AND ANTIOXIDANTS

Strong evidence exists that generation of placental oxidative stress is a key intermediary event in the pathology of preeclampsia (Hubel, 1999; Redman and Sargent, 2000; Burton and Jauniaux, 2004). It is thought that deficient conversion of the uterine spiral arteries and subsequent impaired perfusion of the placenta provides the initiating insult (Brosens et al., 2002). In early gestation of normal pregnancy, the spiral arteries undergo substantial remodeling by invasive extravillous trophoblasts, which penetrate into the myometrium and convert muscular spiral arteries into flaccid tubes with no muscularis or elastic lamina, capable of supplying the hugely expanded blood flow of the third trimester placenta. In contrast, the remodeling is minimal in PE, only affecting the decidual segments of the spiral arteries, which retain their high-resistance (Brosens et al., 2002). Maternal blood thus enters the intervillous space at a higher pressure and a faster rate, in a pulsatile jet-like manner, exposing placental villi to fluctuating oxygen concentrations, which contributes to the ischemia-reperfusion (I/R) type injury of the placenta (Jauniaux et al., 1994, 1995; Burton and Hung, 2003). The resulting oxidative stress is thought to induce the placenta to release a mixture of factors, including inflammatory cytokines, antiangiogenic factors, and apoptotic debris, which culminates in an enhanced maternal inflammatory response and endothelial dysfunction (Roberts, 1998; Redman and Sargent, 2005).

The role of oxidative stress in mediating PE pathophysiology is supported by reports of significantly decreased levels of antioxidant vitamins C, A, E, β-carotene, glutathione levels, and iron-binding capacity in the maternal circulation of women with preeclampsia. There is also evidence of diminished superoxide dismutase (SOD) levels and activity in the maternal and placental compartments of preeclamptic women, indicative of decreased total antioxidant protective capacity in preeclampsia (Walsh, 1998). In the experimental model of reduced uterine perfusion pressure (RUPP) of placental ischemia-induced hypertension, treatment with vitamins C and E did not decrease blood pressure, while the SOD mimetic Tempol attenuated RUPP hypertension. Interestingly, treatment with an NADP(H) oxidase inhibitor attenuated but did not normalize hypertension, suggesting other ROS-generating pathways (Gilbert et al., 2008; Sedeek et al., 2008). Challenging placental explants with hypoxia-reoxygenation (HR) or hypoxia *in vitro* induced sFlt-1 expression via HIF-1α upregulation (Ahmad and Ahmed, 2004; Nagamatsu et al., 2004; Cindrova-Davies et al., 2007). We showed that administration of vitamins C and E can block HR-mediated sFlt-1 secretion, via inhibition of p38 and NF-κB signaling pathways (Cindrova-Davies et al., 2007; Cindrova-Davies, 2009). Similarly, the *in vivo* challenge of labor results in increased oxidative stress, and increased expression of HIF-1 α, sFlt-1 and VEGF (Cindrova-Davies et al., 2007).

The regulatory role of oxidative stress in the I/R-type injury in preeclampsia and cardiovascular disease, together with encouraging *in vitro* and *ex vivo* data introduced the hypothesis that antioxidant vitamins might play an important role in the treatment of the ischemic disease pathology. However, despite encouraging *in vitro* data and cohort trials, clinical trials aimed to treat women at risk of preeclampsia with vitamins C and E (Poston et al., 2006; Rumbold et al., 2006) showed no reduction in the incidence of the disease. These results are disappointing. Periconceptional use of vitamins in women is associated with lower rates of severe preterm births and extreme SGA, which seems to suggest that vitamin use might be effective during conception and early pregnancy (Catov et al., 2007), and that the clinical trial administration of vitamins occurred too late in pregnancy to reverse already established disease pathology. The outcome could also be affected by the composition of natural vs. synthetic vitamins, as evidence exists that healthy Mediterranean diet lowers the incidence of preterm birth (Haugen et al., 2008) and coronary heart disease (Aravanis et al., 1970). Synergy between different mediators may also be important in disease treatment. Recent clinical trial found that whilst antioxidant vitamins alone did not have a protective effect for prevention of PE, there was a synergistic effect between L-arginine and vitamin C, reducing the incidence of PE in these patients (Vadillo-Ortega et al., 2011). Endogenous NADPH oxidase and manganese SOD are required to maintain VEGF signaling and vascular homeostasis (Abid et al., 2001, 2007). Interestingly, despite no effect of the vitamin trials on lowering the incidence of preeclampsia, antioxidant supplementation led to a significant decrease in the plasma concentration of sFlt-1 and an increase in the plasma concentration of PlGF (Poston et al., 2011), matching our *in vitro* findings and confirming the beneficial effect of antioxidants on VEGF signaling. Antioxidant vitamins could interfere with the normal physiological roles of VEGF and there seems to be a scope for potential use of antioxidants in preventing or ameliorating preeclampsia.

## NO SUPPLEMENTATION

Endothelium plays a crucial role in regulating vascular tone. In preeclamptic patients, endothelial activation is manifested by increased expression of markers of endothelial activation, including VCAM, endothelin, von Willebrand Factor, and thrombomodulin (Mutter and Karumanchi, 2008). Nitric oxide (NO), hydrogen sulfide (H_2_S), and carbon monoxide (CO) are three gaseous vasodilators that maintain the vascular tone. NO release from endothelial cells counter-balances the vasoconstriction produced by the sympathetic nervous system and the renin–angiotensin system. In addition, NO exerts many vasoprotective and anti-atherosclerotic properties, including protection from thrombosis, reduction of adhesion molecule expression and leukocyte adhesion. NO is produced by nitric oxide synthase (NOS), using L-arginine as a substrate. In addition, endogenously produced methylated amino acids such as asymmetric dimethylarginine (ADMA) can act as competitive inhibitors, and their secretion is increased in patients with cardiovascular disease and renal failure (Schnabel et al., 2005), as well as in women with high resistance placental circulation at risk of preeclampsia, IUGR, or both (Savvidou et al., 2003).

NO formation was found to be impaired in women with preeclampsia and gestational hypertension, compared to healthy pregnant controls. A negative correlation exists between plasma nitrite levels and sFlt-1 and sEng, suggesting an inhibitory effect of the angiogenic factors on NO formation (Sandrim et al., 2008). Given the important role of NO in the pathophysiology of preeclampsia, much research has concentrated on targeting the NO pathway as a potential therapy for preeclampsia. These include organic nitrates and *S*-nitrosothiols (e.g., *S*-nitrosoglutathione), L-arginine, inhibitors of cGMP breakdown (e.g., sildenafil), and other novel inhibitors of NO donor metabolism. This topic has been reviewed extensively by Johal et al. (2013). *S*-nitroglutathione (GSNO), endogenous *S*-nitrosothiol found ubiquitously in tissue, has been infused in women with preeclampsia and it has been shown to target not only the endothelial dysfunction, but also reduce platelet aggregation and activation, reduce sEng, and improve utero-placental perfusion with no adverse side effects, making it a good potential candidate for PE therapy (de Belder et al., 1995; Lees et al., 1996; Johal et al., 2013). The effects of supplementation with NO precursor, L-arginine, have been studied in several clinical trials. A recent randomized control trial of high risk pregnant women showed that dietary supplementation with a combination of L-arginine and antioxidants was associated with a significant reduction in the incidence of preeclampsia, compared to antioxidants alone or placebo prevention of preeclampsia (Vadillo-Ortega et al., 2011). These are encouraging results although they have to be interpreted with caution, given that the effects of L-arginine alone were not studied, and as such it is difficult to dissect out the relative contributions of L-arginine and antioxidants in reducing the incidence of the disease. These encouraging results were echoed in a recent systematic review of randomized trials focused on the role of L-arginine in the prevention of preeclampsia (Dorniak-Wall et al., 2014). The authors analyzed 7 randomized controlled trials testing the effects of L-arginine in pregnant women, and they reported L-arginine supplementation was associated with a significant reduction in the risk of preeclampsia in pregnant women with either established hypertension or who were considered at risk of preeclampsia (Dorniak-Wall et al., 2014).

The mechanism of the beneficial effect of L-arginine supplementation has been studied in pregnant rats in whom preeclampsia-like phenotype was induced with sFlt-1 administration (Murphy et al., 2011). sFlt-1 infusion into pregnant rats induced hypertension associated with reductions in circulating levels of VEGF, significant proteinuria, and endothelial dysfunction, as marked by a 3.5% increase in renal cortical pro-ET mRNA expression (Maynard et al., 2005; Gilbert et al., 2007; Murphy et al., 2010, 2011). Administration of L-arginine decreased sFlt-1 hypertension but had no effect on the blood pressure response in non-pregnant rats. In addition, L-arginine abolished the sFlt-mediated expression of renal cortical prepro-ET, suggesting that a reduction in NO synthesis may play an important role in the enhanced ET-1 production in response to sFlt-1 hypertension in pregnant rats (Murphy et al., 2011). These data are supported by previous studies, which showed a link between reduced NO production and enhanced ET-1 production (Kourembanas et al., 1993; Edwards et al., 1996). It has been postulated that increased circulating levels of sFlt-1 lead to reduction of VEGF and NOS, thus reducing NO production (George and Granger, 2011).

## ER STRESS AND ER STRESS INHIBITORS

Endoplasmic reticulum (ER) processes all secreted proteins, facilitating folding and post-translational modifications. Accumulation of misfolded proteins leads to activation of ER stress response pathways, collectively known as the Unfolded Protein Response (UPR). The aim of the UPR is to restore homeostasis by inhibiting translation of non-essential proteins, increasing the capacity of the chaperone and folding machinery, and stimulating degradation of misfolded proteins. Failure of the mechanism results in activation of apoptotic pathways. ER stress is a major regulator of cell homeostasis (Xu et al., 2005; Cullinan and Diehl, 2006; Yoshida, 2007). The UPR is initiated by three transmembrane sensors normally held inactive by binding of the principal ER chaperone, GRP78. These sensors are PERK (PRKR-like ER kinase) that phosphorylates eukaryotic initiation factor 2α (eIF2α) to inhibit translation, ATF6 (activating transcription factor 6) and IRE1 (inositol-requiring enzyme 1). ATF6 is cleaved to produce an active transcription factor, while IRE1 splices the mRNA encoding XBP-1 (X-box binding protein-1) to produce a second transcription factor. These factors have overlapping functions, and both upregulate ER chaperone proteins, including GRP78, and degradation pathways (Xu et al., 2005; Cullinan and Diehl, 2006; Yoshida, 2007). In addition, ER stress can generate and accumulate ROS, thus promoting oxidative stress. Activation of the NF-κB pathway links increased oxidative stress, ER stress and inflammation. NF-κB can be activated by an increase in ER stress via PERK-eIF2α activation or IRE1α autophosphorylation. This effect can be blocked by both calcium chelators and antioxidants, suggesting NF-κB activation could be a result of the oxidative stress arising from excessive protein folding and/or ER-stress mediated Ca^2^^+^ leakage (Zhang and Kaufman, 2008).

Disorders of ER function are recognized to underlie many diverse pathologies, including diabetes and neurodegenerative diseases, and therapeutic interventions targeting it are being devised (Hetz et al., 2013). ER stress also seems instrumental in the pathophysiology of preeclampsia and IUGR (Yung et al., 2008, 2012). Use of ER chaperones proved a beneficial therapy for treatment of type 2 diabetes as it restored systemic insulin sensitivity, normalized hyperglycemia, resolved fatty liver disease, and enhanced insulin action in various tissues (Ozcan et al., 2006). Similarities exist between preeclampsia and metabolic syndrome, including dyslipidemia, inflammation, insulin resistance, oxidative stress and ER stress. We have demonstrated an increased expression of ET-1 and sFlt-1 in HR-treated placental explants (Cindrova-Davies et al., 2007; Cindrova-Davies, 2009). Conditioned medium from HR explants induced ER stress in JEG-3 cells, which was abolished by an ET-1 neutralizing antibody (Jain et al., 2012). Administration of ER chaperone TUDCA to placental explants challenged with HR reduced the levels of endothelin-1 (ET-1) in these explants, and it reduced the protein levels of sFlt-1 (unpublished observations). These data suggest that restoring ER function could also offer a therapeutic benefit for the treatment of preeclampsia. The ER chaperones are endogenously produced bile salts ursodeoxycholic acid (UDCA, TUDCA). The chaperones have excellent *in vivo* safety profiles and their use has been approved in clinical trials for the treatment of urea-cycle disorders, thalassemia and cystic fibrosis (Ozcan et al., 2006), and they are currently used to treat intrahepatic cholestasis of pregnancy (Geenes and Williamson, 2009).

## STATINS

Preeclampsia has been associated with dyslipidemia, and there is evidence of increased antibodies for oxidized form of LDL in patients with preeclampsia, which is consistent with oxidative stress, and it is analogous to changes described in atherosclerosis (Branch et al., 1994). Although preeclampsia is unique to pregnancy, there are many biological and pathological similarities, as well as risk factors (e.g., hypertension, obesity, dyslipidemia, diabetes, etc.) with adult cardiovascular disease. The mechanism underlying both atherosclerosis (Hansson, 2005) and preeclampsia (Roberts and Cooper, 2001; Redman and Sargent, 2005) is initiated by underlying endothelial dysfunction and inflammation, which lead to disease progression and manifestation. Pregnancy is often viewed as a stress test, and preeclampsia an early manifestation of CV disease. In fact, women who develop preeclampsia have a two- to threefold risk of hypertension, ischemic stroke and heart disease in later life (van Pampus and Aarnoudse, 2005; McDonald et al., 2008), and the risk is further increased with the severity of the disease, and early manifestation before 34 weeks gestation (Mongraw-Chaffin et al., 2010). Given the analogies between the two diseases, some remedies and treatments used to prevent and treat cardiovascular disease have been tested in preeclampsia.

Natural and synthetic statins (or 3-hydroxy-3-methyl-glutaryl-coenzyme A (HMG-CoA) reductase inhibitors) are the most commonly prescribed classes of medication worldwide, used in primary and secondary prevention of cardiovascular mortality and other cardiovascular events through lipid-lowering remedy, as well as many pleiotropic effects, including endothelial protection, antioxidant properties, anti-inflammatory, antithrombotic and proangiogenic effects (Costantine and Cleary, 2013; Girardi, 2014). The beneficial effects of statins in the treatment of cardiovascular disease have been confirmed by recent meta-analysis of individual data from 90,000 individuals in 14 randomized trials of statin therapy vs. placebo, called the Cholesterol Treatment Trialists’ (CTT) Collaboration (Baigent et al., 2005). The CTT concluded that statin therapy can safely reduce the 5-year incidence of major coronary events, coronary revascularization, and stroke by about one fifth per mmol/L reduction in LDL cholesterol, largely irrespective of the initial lipid profile or other presenting characteristics (Baigent et al., 2005).

The effect of pravastatin administration in pregnancy has been tested in many rodent models of preeclampsia. Treatment with pravastatin significantly reduced maternal sFlt-1 levels, lowered blood pressure, improved the vascular profile, and prevented kidney injury (Ahmed et al., 2010; Costantine et al., 2010; Fox et al., 2011; Kumasawa et al., 2011; Saad et al., 2014). In addition, pravastatin also prevented the incidence of intrauterine growth restriction, without any adverse effects on pregnancy. In a mouse model of preeclampsia using placenta-specific lentiviral vector expression of sFlt-1, Kumasawa et al. elegantly demonstrated that pravastatin administration decreased sFlt-1 but importantly increased PlGF, thus restoring the angiogenic balance (Kumasawa et al., 2011). Pravastatin effects could be reproduced by administration of PlGF, which reduced sFlt-1 levels, ameliorated hypertension, glomerular endotheliosis, and proteinuria in the mice, suggesting that the beneficial effect of pravastatin on improving preeclampsia-like symptoms is mediated by increasing PlGF levels, which counteracts sFlt-1-mediated effects (Kumasawa et al., 2011). In addition, statins also exert protective effects on the endothelium and ameliorate preeclampsia symptoms by increasing the release of vasodilators NO (Redecha et al., 2009; Fox et al., 2011) and CO (Cudmore et al., 2007; Muchova et al., 2007). Pravastatin treatment of mice destined to develop preeclampsia using sFlt-1 overexpression increased eNOS protein expression in the vasculature (Fox et al., 2011), whilst pravastatin improved pregnancy outcome by increasing plasma NO levels in CBAxDBA/2 mice destined to develop recurrent miscarriage and IUGR (Redecha et al., 2009). Treatment of mice with statins resulted in increased HO-1 activity and increased CO release from tissues, as well as increased levels of plasma antioxidants (Muchova et al., 2007). Statins also induced the expression of HO-1 and inhibited sFlt-1 secretion in placental explants (Cudmore et al., 2007). These studies provide further evidence of a pleiotropic role of pravastatin in preventing the vascular dysfunction.

Given the encouraging evidence of the beneficial effects of statins on the prevention of cardiovascular disease in humans and of preeclampsia in animal models, the ability of pravastatin to restore angiogenic balance is currently being tested in the first randomized placebo controlled StAmP trial (Statins to ameliorate early onset preeclampsia) in the UK. The aim of the trial is to establish if pravastatin can lead to a significant reduction in circulating antiangiogenic factors and alleviate the severity of early onset preeclampsia. The pleiotropic anti-inflammatory, anti-thrombotic, antioxidant and vascular protective effects suggest that statins might be a good therapeutic option to prevent preeclampsia.

## FUTURE DIRECTIONS – H_2_S DONORS

Endogenous H_2_S plays an important role in regulating physiological processes such as blood flow, vasodilation, arterial diameter, and leukocyte adhesion (Wang, 2009). Endogenous production is catalyzed by cystathionine β-synthase (CBS) and cystathionine γ-lyase (CSE). H_2_S is produced in the cardiovascular system by CSE, using cysteine as a substrate. It acts as a vasodilator, being able to hyperpolarize and relax SMCs by opening K_ATP_ channels (Zhao et al., 2001). Unlike NO and CO, which mediate vasorelaxation largely by cGMP pathway activation, the vasorelaxant effect of H_2_S is independent of cGMP pathway (Zhao et al., 2001). In addition, NO effects depend largely on the endothelial function integrity, whereas H_2_S is an endothelium-independent vasodilator and exerts its direct effect on SMCs. CSE knockout mice have markedly reduced serum H_2_S levels, which results in pronounced hypertension and diminished vasodilation, providing direct evidence that H_2_S is a potent vasodilator that can influence blood pressure (Yang et al., 2008). Similarly, genetic deficiency of CBS leads to homocysteinemia, which is associated with endothelial dysfunction and hypertension (Miles and Kraus, 2004). We reported reduced CSE expression in IUGR cases, as well as PE cases that were accompanied by abnormal UA Doppler profiles, and this could be recapitulated by subjecting placental explants to HR. In addition, we demonstrated a potent vasodilatory effect of an H_2_S donor in perfused placentas, capable of K_ATP_ dependent-reduction of placental vascular resistance. Reduced bioavailability of H_2_S may thus be implicated in placental vasoconstriction (Cindrova-Davies et al., 2013). In addition to being a potent vasodilator, many animal studies also showed a protective role of H_2_S donors against ischemia-reperfusion injury and inflammation (Bos et al., 2009, 2013; Sodha et al., 2009). The pro-survival pathways AKT, PKC, and ERK1/2 have been identified as H_2_S targets conferring its anti-apoptotic effects during reperfusion injury (Hu et al., 2008; Yong et al., 2008). Additionally, H_2_S can promote direct anti-apoptotic signaling during ischemia-reperfusion by activating eNOS and thus inducing NO (Yong et al., 2008), and H_2_S promotes DNA binding and transcriptional activation of anti-apoptotic genes regulated by NF-κB (Sen et al., 2012). The antioxidant properties of H_2_S can be mediated via two distinct mechanisms; by acting as a direct scavenger of ROS and by up-regulating antioxidant defenses. H_2_S may up-regulate endogenous antioxidants through a nuclear factor E2-related factor-2 (Nrf2; Calvert et al., 2009). Nrf2 binds the antioxidant responsive element (ARE) found in the promoter region of antioxidant genes, including heme oxygenase-1 (HO-1), thioredoxin, thioredoxin reductase, glutathione reductase, glutathione peroxidase (GPx), glutathione *S*-transferase (GSS), and catalase and is thus an important factor in controlling cardiac cellular susceptibility to reactive oxygen and nitrogen species-induced cytotoxicity (Zhu et al., 2005). Garlic oil can also promote Nrf-2 activation, further supporting the role for Nrf2 as the mediator of H_2_S-induced antioxidant effects (Fisher et al., 2007). Additionally, CSE expression appears to be a critical component of the cytoprotective ATF4 transcriptional response; CSE deficiency increases sensitivity to apoptosis induced by ER stressors and homocysteine, indicating the importance of GSH up-regulation through the transsulfuration pathway to promote survival (Dickhout et al., 2012). H_2_S also exerts important proangiogenic effects (Cai et al., 2007; Wang et al., 2010; Bir et al., 2012; Holwerda et al., 2014). H_2_S promotes proliferation, adhesion, migration and tube-like structure formation of endothelial cells *in vitro* via AKT pathway phosphorylation. It also stimulates angiogenesis *in vivo* at physiologically relevant doses (Cai et al., 2007). NO-H_2_S cross-talk seems critical in mediating these effects (Bir et al., 2012; Coletta et al., 2012). H_2_S therapy restores blood flow to ischemic tissues in a NO-dependent manner, by stimulating NOS expression and HIF-1α and VEGF expression and activity (Bir et al., 2012).

Consistent with these roles of H_2_S, the consumption of garlic is negatively correlated with the progression of CV disease, and causes a lower incidence of hypertension, enhances antioxidants and inhibits platelet aggregation (Banerjee and Maulik, 2002). It has been shown that the biological production of H_2_S from garlic-derived organic polysulfides mediates the major beneficial effects of garlic-rich diets, specifically on CV disease and more broadly on overall health (Benavides et al., 2007). In animal models of hypoxic pulmonary hypertension, H_2_S could reverse structural remodeling changes in pulmonary vessels of rats exposed to chronic HR (Hongfang et al., 2006). H_2_S therapy also protected against acute myocardial ischemia/reperfusion (Calvert et al., 2009), and attenuated cardiac dysfunction following heart failure (Kondo et al., 2013; Polhemus et al., 2013). Recent animal studies have focused on the use of “medical food,” i.e., drugs derived from natural sources such as garlic, as a cardioprotective therapy for heart failure. These include diallyl trisulfide (DATS; Polhemus et al., 2013), a long acting H_2_S donor derived from garlic, and sulfur-releasing “medical food” SG-1002 (Kondo et al., 2013), which is currently in clinical trials. Similar results have been reported in the placental field recently. Holwerda et al. (2014) evaluated the therapeutic effect of an H_2_S donor in an animal model of sFlt-induced hypertension. They reported a significant reduction in blood pressure, and proteinuria, as well as concomitant reduction in sFlt-1 levels and increase in free maternal VEGF concentrations in the treated group of animals (Holwerda et al., 2014). Wang et al. (2013) demonstrated that CSE enzyme inhibition in pregnant mice induced hypertension, increased sFlt-1 and sEng levels, caused placental abnormalities and compromised fetal growth. H_2_S donor therapy reduced the levels of the antiangiogenic factors and restored fetal growth, adding further weight to the evidence that a dysfunctional CSE/H_2_S pathway may contribute to the pathogenesis of preeclampsia (Wang et al., 2013), and that H_2_S donor therapy could have beneficial effects on prevention of preeclampsia.

## CONCLUDING REMARKS

**FIGURE 1:**
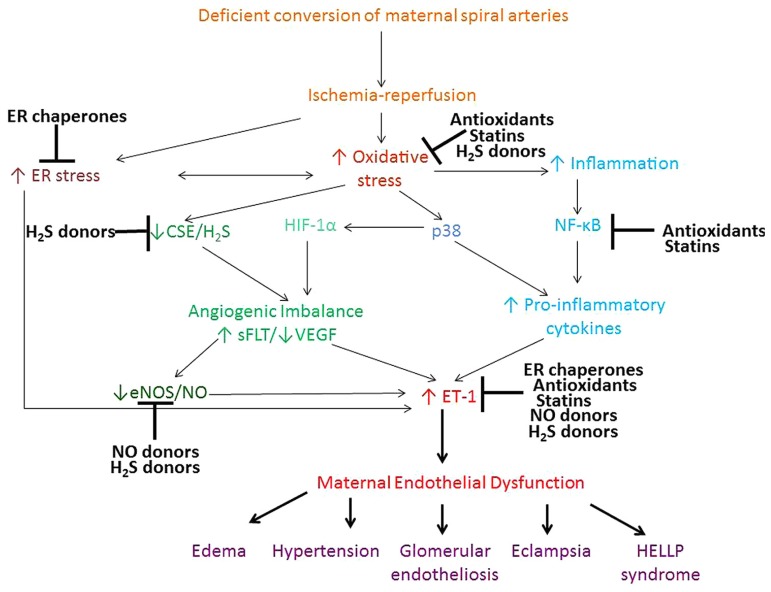
**Proposed pathways contributing to the maternal syndrome of preeclampsia and the mechanism of action of antioxidants, ER chaperones, NO, and H_2_S donors and statins**. Deficient conversion of spiral arteries leads to fluctuations in O_2_ concentration and triggers ischemia-reperfusion type injury of the placenta and consequent oxidative stress and ER stress. ROS activate various stress pathways, including proapoptotic p38 and inflammatory NF-κB pathway. These pathways promote increased shedding of microparticles, anti-angionenic factors and inflammatory cytokines, which lead to development of the peripheral maternal symptoms. Antioxidants, ER chaperones, NO and H_2_S donors and statins all display pleitrophic effects, targeting inflammatory, stress, antioxidant pathways, as well as VEGF and NO signaling.

Therapeutic interventions to treat preeclampsia remain largely experimental and there is no established remedy for the treatment of this multifactorial disease. This review concentrated on the evidence for the therapeutic potential of antioxidants, ER chaperones, NO and H_2_S donors, and statins. Mechanistic effects of these compounds on the signaling pathways involved in the pathophysiology of preeclampsia, are summarized in **Figure 1**. Antioxidants, ER chaperones, NO donors, statins and H_2_S donors display pleitropic antioxidant, anti-inflammatory, and pro-angiogenic effects in animal and *in vitro* studies. Encouraging clinical evidence exists for the use of NO donors, and a clinical trial is currently underway testing the effect of statins in treatment of preeclampsia. Although clinical trials on the use of antioxidant vitamins in pregnancy proved largely unsuccessful, the scope for their use still exists given the beneficial cardioprotective effects of antioxidant-rich Mediterranean diet, periconceptual vitamin use and the synergistic effect of vitamin C and L-arginine. H_2_S recently emerged as a novel therapeutic agent for cardiovascular disease and its effects are being tested.

## Conflict of Interest Statement

The author declares that the research was conducted in the absence of any commercial or financial relationships that could be construed as a potential conflict of interest.

## ABBREVIATIONS

ADMA: asymmetric dimethylarginine
AKT: serine/threonine-specific protein kinase
ALK1 and ALK5: TGF-β receptors type I
ARE: antioxidant responsive element
ATF6: activating transcription factor 6
CBS: cystathionine β-synthase
CO: carbon monoxide
CSE: cystathionine γ-lyase
CTT: cholesterol treatment trialists
CV: cardiovascular
DAMPs: damage-associated molecular pattern
EDF: end diastolic flow
eIF2α: eukaryotic initiation factor 2α
eNOS: endothelial nitric oxide synthase
ER: endoplasmic reticulum
ERK: extracellular signal-regulated kinase
ET-1: endothelin-1
GPx: glutathione peroxidase
GRP78: 78 kDa glucose-regulated protein, also known as binding immunoglobulin protein (BiP) or heat shock 70 kDa protein 5 (HSPA5)
GSS: glutathione S-transferase
H_2_S: hydrogen sulfide
HELLP syndrome: hemolysis, elevated liver enzymes and low platelets
HO-1: heme oxygenase
HIF-1α: hypoxia-induced factor
HR: hypoxia-reoxygenation
ICAM: intercellular adhesion molecule
I/R: ischemia-reperfusion
IRE1: inositol-requiring enzyme 1
IUGR: intrauterine growth restriction
LDL: low density lipoprotein
NADPH: nicotinamide adenine dinucleotide phosphate
NF-κB: nuclear factor kappa-light-chain-enhancer of activated B cells
NO: nitric oxide
Nrf2: nuclear factor E2-related factor-2
p38 MAPK: p38 mitogen-activated protein kinase
PE: preeclampsia
PERK: PRKR-like ER kinase
PlGF: placental growth factor
ROS: reactive oxygen species
RUPP: reduced uterine perfusion pressure
sEng: soluble TGF-β co-receptor endoglin
sFlt1: soluble VEGF receptor 1, also referred to as soluble fms-like tyrosine kinase
SGA: small for gestational age
SOD: superoxide dismutase
StAmP trial: Statins to ameliorate early onset preeclampsia
TLR: Toll-like receptors
TUDCA: Tauroursodeoxycholic acid, bile salt
UDCA: ursodeoxycholic acid, bile salt
UPR: Unfolded Protein Response
VCAM-1: vascular cell adhesion molecule 1
VEGF: vascular endothelial growth factor
XBP-1: X-box binding protein-1.
